# Manifestation of anaplasmosis as cerebral infarction: a case report

**DOI:** 10.1186/s12879-018-3321-4

**Published:** 2018-08-17

**Authors:** Seok Won Kim, Choon-Mee Kim, Dong-Min Kim, Na Ra Yun

**Affiliations:** 1 Department of Neurosurgery, College of Medicine, Chosun University, Gwangju, Republic of Korea; 2 Premedical Science, College of Medicine, Chosun University, Gwangju, Republic of Korea; 3 Departments of Internal Medicine, College of Medicine, Chosun University, 588 Seosuk-dong, Dong-gu, Gwangju, 61453 Republic of Korea

**Keywords:** Anaplasmosis, Cerebral infarction, Human granulocytic anaplasmosis and Anaplasma phagocytophilum

## Abstract

**Background:**

Human granulocytic anaplasmosis is a tick-borne zoonotic disease caused by *Anaplasma phagocytophilum*, an obligate intracellular granulocytotropic bacterium.

**Case presentation:**

A 70-year-old female patient was admitted with the clinical signs of fever and an altered state of consciousness 1 week after experiencing a tick bite while planting lawn grass. Magnetic resonance imaging, performed at the time of admission, indicated cerebral infarction in the left basal ganglia, whereas increasing immunofluorescence assay antibody titers for *A. phagocytophilum* were also documented. *A. phagocytophilum* was identified using *groEL* and *ankA* targeted polymerase chain reaction and sequencing. Because of severe thrombocytopenia, only doxycycline was administered, without any antiplatelet agents. Subsequently, the symptoms improved without any focal neurologic sequela.

**Conclusion:**

This is the first reported case of cerebral infarction occurrence in an anaplasmosis patient.

## Background

Human granulocytic anaplasmosis (HGA) refers to an acute febrile disease caused by the bite of the tick species *Ixodes* infected with *Anaplasma phagocytophilum*. In this condition, patients complain of various non-specific symptoms, including fever, chills, headache, and muscle ache [1]. Additionally, a wide range of symptoms, including vertigo, upper gastrointestinal bleeding, seizure, and confusion, have been reported in HGA patients [2].

Previously, there has been a reported case of cerebral infarction caused by macrovascular involvement in association with Mediterranean spotted fever caused by *Rickettsia conorii* [3], as well as cerebral hemorrhage or cerebral infarction in association with scrub typhus [4].

In general, the primary host cells of *A. phagocytophilum* are the granulocytes, particularly neutrophils. Previous studies have also demonstrated the infection in endothelial cells [5].

However, there have been no reported cases of cerebral infarction in HGA or ehrlichiosis patients to date. Here, we present a case of cerebral infarction that occurred in an HGA patient who was admitted for symptoms of fever and an altered state of consciousness after a tick bite.

## Case presentation

A 70-year-old female patient was admitted for confusion. One week before admission, the patient discovered, while bathing, that she had been bitten by a tick that had attached to her skin while she was planting lawn grass earlier, and removed the tick accordingly. She reported that the tick was approximately 3 mm in size, but that she disposed of the tick after removal. Subsequently, there were no specific symptoms and she continued in planting lawn grass. However, 3 days after the tick bite, she began to develop dizziness with a fever. On the day of admission, she was waving her hands, was non-communicative, and provided irrelevant responses to questions. She appeared relatively fine while lying down, but, when standing up, the symptoms became severe and she struggled to maintain her balance. Therefore, she was admitted to the emergency room for further evaluation.

**Fig. 1:**
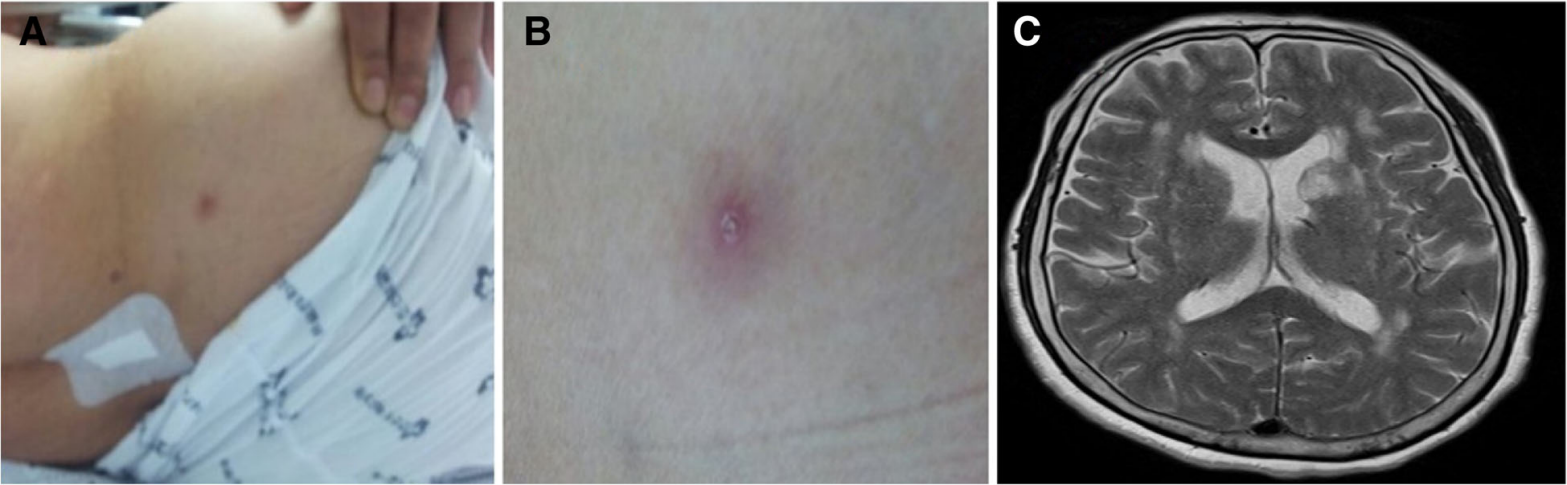
A 70-year-old female patient with a confirmed diagnosis of human granulocytic anaplasmosis. **a** A lesion suspected of being a tick bite found on the right buttocks area at the time of admission. **b** Magnified image of the site of suspected tick bite. **c** Right middle cerebral artery territory infarction (recent onset infarction)on T2 -weightedimage on brain magnetic resonance imaging

At the time of admission, a lesion suspected of being a tick bite with a diameter of approximately 5 mm was found in the right buttocks area (Fig. 1). Her blood pressure, pulse, respiratory rate, and body temperature at admission were 100/60 mmHg, 88 beats/min, 24 breaths/min, and 38 °C, respectively. Blood test results indicated a white blood cell (WBC) 920 /μL (neutrophil 86.8%), hemoglobin level of 14.1 g/dL, and platelet level of 22,000/μL, whereas the biochemistry test results indicated: aspartate aminotransferase 99.9 IU/L, alanine transaminase 54.7 IU/L, total bilirubin 1.3 mg/dL, blood urea nitrogen 20.1 mg/dL, creatinine 0.67 mg/dL, cholesterol 156 mg/dL, and triglyceride 81 mg/dL. Although the erythrocyte sedimentation rate was 7 mm/h, the C-reactive protein level was increased to 22 mg/dL, whereas lactate dehydrogenase and creatine phosphokinase levels were elevated to 1052 (normal: 200–450) U/L and 1394 (normal: 55–215) U/L, respectively. The blood coagulation test showed normal findings in prothrombin time (10.7 s), international normalized ratio (0.96), activated partial thromboplastin time (26 s), and fibrinogen (384 mg/dL), but elevated levels of fibrinogen degradation products (50.3 [normal: 0–5.0] μg/mL) and D-dimer (3199 [normal: 0–255] ng/mL). A lacunar infarction in the left basal ganglia was found during magnetic resonance imaging for determining the cause of the altered state of consciousness at the time of admission (Fig. 1); however, no significant stenosis or occlusion was found on magnetic resonance angiography. A cerebrospinal fluid (CSF) tap was performed to rule out encephalitis and meningitis, although there was no neck stiffness, with the results showing CSF WBC 0 per mm^3^, CSF protein 34.7 mg/dL, and glucose 130.2 mg/dL (serum glucose 221.3 mg/dL). No microorganisms were found in cultured blood and CSF using the BACTEC culture system (Becton Dickinson, Towson, MD, USA), whereas cerebral fluid herpes virus, enterovirus, *Orientia tsutsugamushi,* and *Leptospira interrogans* polymerase chain reaction all tested negative.

Although cerebral infarction in the left basal ganglia was identified, the patient exhibited low levels of platelets. Therefore, she was not qualified to receive antiplatelet agents. Furthermore, based on clinical signs of fever and altered state of consciousness after a tick bite, doxycycline 100 mg twice daily was administered starting from post-admission day 2. The fever began to subside 1 day after doxycycline administration and resulted in the rapid resolution of symptoms. The patient showed a complete recovery of her consciousness by the 4th day of doxycycline administration.

**Fig. 2:**
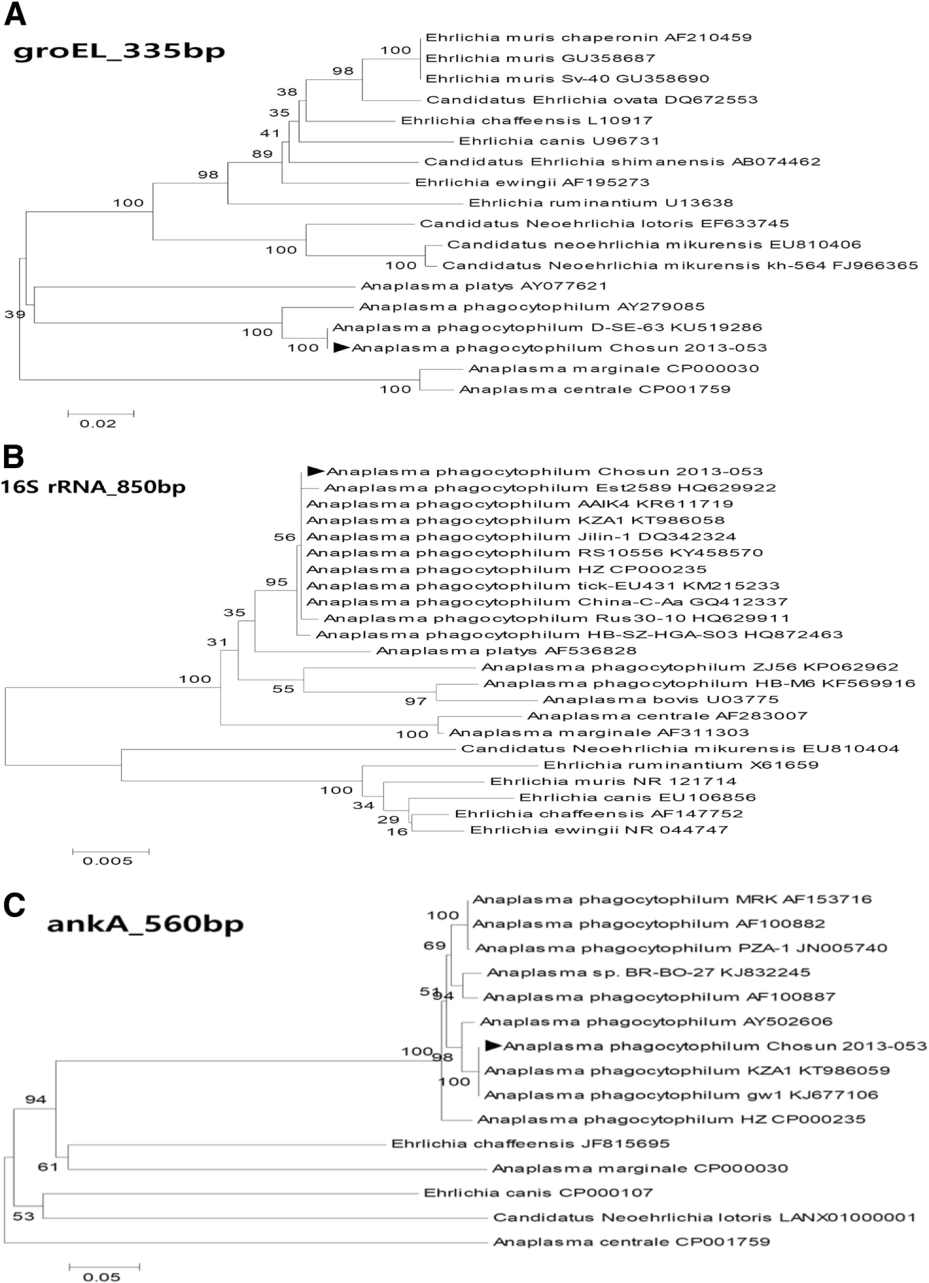
Polymerase chain reaction (PCR) phylogenetic trees. The trees were generated using the buffy coat of a 70-year-old female human granulocytic anaplasmosis patient admitted for the chief complaint of fever. **a** Phylogenetic tree after *groEL* nested PCR. **b** Phylogenetic tree after *16S rRNA* nested PCR. **c** Phylogenetic tree after *AnkA* nested PCR

A blood sample was tested using nested PCR with *Anaplasma* and *Ehrlichia-*specific primers targeting the GroEL heat-shock protein gene (*groEL*) and ankyrin-repeat protein AnkA gene (*ankA*), Additionally, a portion of the 16S ribosomal RNA gene (16S rRNA) was amplified using PCR [6–8]. The PCR amplicons were purified and directly sequenced using PCR primers. BLAST (Basic Local Alignment Search Tool) analysis of sequenced productsconfirmed *A. phagocytophilum* infection (Fig. 2), although morulae were not detected in a stained peripheral blood smear.

Immunofluorescence assay (IFA) antibodies against *A. phagocytophilum* were also measured from the blood sample [8]. Upon admission, the immunoglobulin (Ig) M level was below 1:16 and the IgG level was below 1:80. At 7 days later, the IgM level was 1:64 and the IgG level was 1:320. The IFA examination using CSF showed an IgM level below 1:16 and an IgG level below 1:80.

Furthermore, other blood tests were negative for Hantavirus, severe fever thrombocytopenia syndrome virus, *O. tsutsugamushi*, and leptospirosis. Indirect IFA and reverse transcription-PCR were performed to diagnose hemorrhagic fever with renal syndrome and severe fever with thrombocytopenia syndrome in blood specimens, respectively [9–11]. Scrub Typhus RAPID kit (ImmuneMed, Republic of Korea) and 56-kDa nested PCR were used for the diagnosis of scrub typhus [12]. Leptospira RAPID kit manufactured by ImmuneMed (Republic of Korea) and *hap1* nested PCR were used for the diagnosis of leptospirosis [13].

The patient exhibited an improvement in symptoms after doxycycline treatment and was discharged on the 12th day with no specific sequela.

## Discussion and conclusions

HGA is a tick-borne zoonotic disease caused by *A. phagocytophilum*, an obligate intracellular granulocytotropic bacterium. To date, there have been no reported cases of cerebral infarction in an HGA patient. Such cases of thromboembolism have not been reported even in ehrlichiosis patients who show similar symptoms. However, portal vein and aortic thromboses were identified in a dog that contracted chronic ehrlichiosis with hypothyroidism, in which case improved clinical signs and diminished thrombi were reported after antibiotics treatment [14].

In addition, in a horse experimentally infected with *A. phagocytophilum*, an autopsy performed 2 days after death found widespread hemorrhage in the internal organs, along with vasculitis and thrombosis in the kidneys [15]. Another study in white footed mice showed extensive brain infection by *Ehrlichia muris* [16]; however, until now, there have been no reports on any HGA patients with thrombotic or embolic cerebral infarction. Secondary platelet dysfunction caused by an infectious disease results in platelet activation, coagulation, and, subsequently, prothrombotic state or reduced platelet function, which results in bleeding diatheses [17–19]. Platelets and *A. phagocytophilum* were incubated to cause platelet hypofunction, from which reduced responses to collagen, adenosine diphosphate, and epinephrine were reported. Such platelet dysfunction was dependent on the *A. phagocytophilum* dose, with a higher dose resulting in more severe alteration of function [20].

Autopsies on patients with Rocky Mountain spotted fever found non-occlusive fibrin thrombi in various organs, such as the gastrointestinal tract, pancreas, liver, kidney, and lung, and such thrombi were localized to rickettsia infection and vascular injury sites [5, 20, 21]. For *A. phagocytophilum*, the primary host cells are granulocytes and endothelial cells are also infected [5]. Accordingly, additional studies are deemed necessary to determine whether cerebral infarction occurred due to thrombosis brought on by endothelial cell injury caused by *A. phagocytophilum*, as in other rickettsia cases, or whether cerebral infarction occurred due to platelet dysfunction caused by *A. phagocytophilum*.

In conclusion, this case was the first reported case of cerebral infarction occurring in an anaplasmosis patient. As a clear resolution of clinical signs was achieved after doxycycline administration, anaplasmosis was believed to be the most likely cause of cerebral infarction.

## Abbreviations

CSF: Cerebrospinal fluid
HGA: Human granulocytic anaplasmosis
IFA: Immunofluorescence assay
Ig: Immunoglobulin
PCR: Polymerase chain reaction
WBC: White blood cells
